# Use of Unvalidated Urine Mycotoxin Tests for the Clinical Diagnosis of Illness — United States, 2014

**Published:** 2015-02-20

**Authors:** Melody Kawamoto, Elena Page

**Affiliations:** 1 Division of Surveillance, Hazard Evaluations, and Field Studies, National Institute for Occupational Safety and Health, CDC

In February 2014, CDC’s National Institute for Occupational Safety and Health received a request for a health hazard evaluation from a union representative in an office building. A female employee reported the onset of symptoms involving multiple organ systems upon returning to work after a prolonged absence. The employee searched the Internet for descriptions of symptoms matching hers, found a laboratory offering “toxic mold testing” direct to consumers, and submitted a urine sample, despite the absence of musty odors and signs of fungal growth in her office. The laboratory reported “positive” concentrations of two mycotoxins: ochratoxin at 2.8 parts per billion (ppb) and tricothecenes at 0.4 ppb. The laboratory cutoff for “positive” was ≥2.0 ppb for ochratoxin and ≥0.2 ppb for tricothecenes. The interpretation accompanying the laboratory report said the results “revealed that you have an unusual level of that mycotoxin(s) present in your body.”

The laboratory referred the employee to a clinic specializing in “medical treatment for mold exposure and mold illness,” where she was examined, diagnosed with mold toxicity, and prescribed an antifungal medication. Antifungal medications are used to treat fungal infections, not illnesses caused by toxins produced by fungi. Also prescribed were dietary modification (eating only canned chicken and white rice for 3 days) and several nonstandard medical treatments (e.g., bowel evacuation or hydrocolonic irrigation, cupping therapy, and ionic nasal spray).

Two consultants, one hired by the building manager and one by the employee, carried out destructive testing (removal of drywall, carpet, and ceiling tiles) in the employee’s office. No evidence of water damage or significant fungal growth was found. The cost to the building manager exceeded $25,000. The employee remained convinced that mold exposure occurred in the workplace. Some coworkers, aware of the destructive testing and the urine mycotoxin testing, began to attribute nonspecific symptoms to workplace mold exposures.

The laboratory mentioned its Clinical Laboratory Improvement Amendments (CLIA) certification on its reports and noted that the urine mycotoxin testing was not approved by the Food and Drug Administration (FDA). CLIA regulations require any laboratory that performs testing on patient specimens to have an appropriate CLIA certificate and to meet applicable quality and analytic standards to ensure accurate and reliable test results.* CLIA regulations, however, do not address the clinical validity of testing (i.e., the accuracy with which the test identifies, measures, or predicts a patient’s clinical status).† FDA clearance or approval of a test provides assurance that the test has adequate analytical and clinical validation and that it is safe and effective.§ There is no FDA-approved test for mycotoxins in human urine.

During the past 10 years, CDC’s National Institute for Occupational Safety and Health has received many requests for workplace evaluations based on the results of unvalidated laboratory tests purported to diagnose occupational and environmental illnesses caused by exposure to fungi (including molds). Using unvalidated laboratory tests to diagnose work-related illness can lead to misinformation and fear in the workplace; incorrect diagnoses; unnecessary, inappropriate, and potentially harmful medical interventions; and unnecessary or inappropriate environmental and occupational evaluations (1,2).

Mycotoxins are metabolites of some fungi that can cause illness in humans and animals, primarily after ingestion of contaminated foods. Low levels of mycotoxins are found in many foods; therefore, mycotoxins are found in the urine of healthy persons (3,4). Mycotoxin levels that predict disease have not been established. Urine mycotoxin tests are not approved by FDA for accuracy or for clinical use.

CDC does not recommend biologic testing of persons who work or live in water-damaged buildings nor routine environmental sampling for mold (5,6). To identify possible mold contamination, visual inspection is the first step. To inspect the interior of walls and other difficult-to-examine spaces, a borescope can be inserted through a small hole. Moisture meters can measure moisture in building materials such as carpet, wallboard, wood, brick, and concrete. Identification and elimination of sources of moisture and cleaning or replacement of contaminated materials is essential.

Persons using direct-to-consumer laboratory tests that have not been approved by FDA for diagnostic purposes and their health care providers need to understand that these tests might not be valid or clinically useful. Additional information about molds and their health effects is available at http://www.cdc.gov/mold/faqs.htm#mold.

## Footnotes

* Additional information available at http://www.cms.gov/regulations-and-guidance/legislation/clia/downloads/ldt-and-clia_faqs.pdf.

† Additional information available at 42 U.S.C. §263a; 42 CFR Part 493.

§ Additional information available at 21 U.S.C. §§360c, 360e and 21 CFR 814.20, 860.7.
